# Proteinase-activated receptor 2 (PAR_2_) in hepatic stellate cells – evidence for a role in hepatocellular carcinoma growth *in vivo*

**DOI:** 10.1186/s12943-016-0538-y

**Published:** 2016-07-29

**Authors:** Franziska Mußbach, Hendrik Ungefroren, Bernd Günther, Kathrin Katenkamp, Petra Henklein, Martin Westermann, Utz Settmacher, Lennart Lenk, Susanne Sebens, Jörg P. Müller, Frank-Dietmar Böhmer, Roland Kaufmann

**Affiliations:** 1 Department of General, Visceral and Vascular Surgery, Jena University Hospital, Erlanger Allee 101, D-07747 Jena, Germany; 2 First Department of Medicine, UKSH and University of Lübeck, Lübeck, Germany; 3 Service Unit Small Animal, Research Center Lobeda (FZL), Jena University Hospital, Jena, Germany; 4 Institute of Pathology, Jena University Hospital, Jena, Germany; 5 Institute of Biochemistry, Charité, Berlin, Germany; 6 Electron Microscopy Center, Jena University Hospital, Jena, Germany; 7 Group Inflammatory Carcinogenesis, Institute for Experimental Cancer Research, Christian-Albrechts-University Kiel and University Hospital Schleswig-Holstein (UKSH), Campus Kiel, Kiel, Germany; 8 Institute of Molecular Cell Biology, Center for Molecular Biomedicine, Jena University Hospital, Jena, Germany

**Keywords:** Proteinase-activated receptor 2, PAR_2_, Hepatocellular carcinoma, HCC, Hepatic stellate cells, HSCs, LX-2, Cell migration, Receptor tyrosine kinase, Met, Src, HCC xenograft, Angiogenesis

## Abstract

**Background:**

Previous studies have established that proteinase-activated receptor 2 (PAR_2_) promotes migration and invasion of hepatocellular carcinoma (HCC) cells, suggesting a role in HCC progression. Here, we assessed the impact of PAR_2_ in HCC stromal cells on HCC growth using LX-2 hepatic stellate cells (HSCs) and Hep3B cells as model.

**Methods:**

PAR_2_ expression and function in LX-2 cells was analysed by RT-PCR, confocal immunofluorescence, electron microscopy, and [Ca^2+^]_i_ measurements, respectively. The impact of LX-2-expressed PAR_2_ on tumour growth in vivo was monitored using HCC xenotransplantation experiments in *SCID* mice, in which HCC-like tumours were induced by coinjection of LX-2 cells and Hep3B cells. To characterise the effects of PAR_2_ activation in LX-2 cells, various signalling pathways were analysed by immunoblotting and proteome profiler arrays.

**Results:**

Following verification of functional PAR_2_ expression in LX-2 cells, in vivo studies showed that these cells promoted tumour growth and angiogenesis of HCC xenografts in mice. These effects were significantly reduced when *F2RL1* (encoding PAR_2_) was downregulated by RNA interference (RNAi). In vitro studies confirmed these results demonstrating RNAi mediated inhibition of PAR_2_ attenuated Smad2/3 activation in response to TGF-β1 stimulation in LX-2 cells and blocked the pro-mitotic effect of LX-2 derived conditioned medium on Hep3B cells. Furthermore, PAR_2_ stimulation with trypsin or a PAR_2_-selective activating peptide (PAR_2_-AP) led to activation of different intracellular signalling pathways, an increased secretion of pro-angiogenic and pro-mitotic factors and proteinases, and an enhanced migration rate across a collagen-coated membrane barrier. Silencing *F2RL1* by RNAi or pharmacological inhibition of Src, hepatocyte growth factor receptor (Met), platelet-derived growth factor receptor (PDGFR), p42/p44 mitogen activated protein kinase (MAPK) or matrix-metalloproteinases (MMPs) blocked PAR_2_-AP-induced migration.

**Conclusion:**

PAR_2_ in HSCs plays a crucial role in promoting HCC growth presumably by mediating migration and secretion of pro-angiogenic and pro-mitotic factors. Therefore, PAR_2_ in stromal HSCs may have relevance as a therapeutic target of HCC.

**Electronic supplementary material:**

The online version of this article (doi:10.1186/s12943-016-0538-y) contains supplementary material, which is available to authorized users.

## Background

Hepatocellular carcinoma (HCC) is the fifth most common malignancy worldwide and the second most frequent cause of cancer-related death [1]. Curative liver transplantation or resection is only possible in approximately 30 % of patients. For advanced HCC, the multi-kinase inhibitor sorafenib remains the only approved chemotherapy agent with survival benefits for advanced stage HCC patients. However, its use is limited to those with well-preserved liver function [2–4].

Up to 90 % of cases of HCC develop on the background of advanced liver fibrosis or cirrhosis, thus representing a typical inflammation-related cancer type. It is characterised by a tumour microenvironment where both tumour cells and stromal cells interact via secretion of cytokines, proteinases and other biological active factors. Key players in the organization of an optimal HCC stromal compartment are activated hepatic stellate cells (HSCs) [5, 6]. HSCs are characterised by high proliferation, pronounced matrix proteinase activity and secretion of extracellular matrix (ECM) proteins, as well as of factors including different proteases that aid in creating an immunosuppressive, angiogenic and tumour growth-promoting environment, and thereby critically contribute to HCC initiation and progression [7–18].

Proteinase-activated receptor 2 (PAR_2_) [19] belongs to a group of G protein-coupled receptors (for reviews see: [20–24]). It can be activated by serine proteinases including trypsin, neutrophil proteinase 3, mast cell tryptase, tissue factor(TF)/factor VIIa/factor Xa, human kallikrein-related peptidases and membrane-tethered serine proteinase-1/matriptase 1 as well as by parasite cysteine proteinases [25] and cathepsin S [26], but is insensitive to thrombin [21, 23]. Proteolytic cleavage of PAR_2_ at a specific domain in the extracellular NH_2_-terminus unmasks a “tethered ligand” that interacts with the body of the receptor. In addition, short synthetic peptides based on the proteolytically revealed receptor sequences (PAR_2_-activating peptides; PAR_2_-APs) and chemical modified peptide analogues, such as 2-furoyl-LIGRLO-NH_2_ [22, 27], can activate PAR_2_ in the absence of proteolytic cleavage. Beside a role in regulating physiological responses ranging from vasoregulation and cell growth to inflammation and nociception (reviewed in [20, 21, 23, 28, 29]) there is growing evidence for a function of PAR_2_ in tumours especially from epithelial origin [30–39]. In the setting of liver cancer, it could be demonstrated that PAR_2_ is expressed in HCC tissues, different HCC cell lines and primary HCC cultures established from surgically resected specimens of primary HCCs, where it stimulates liver carcinoma cell migration and invasion via different signalling pathways including [Ca^2+^]_i_ mobilisation, Src, Met and p42/p44 MAPK [40, 41], and is involved in the regulation of CD47^+^ HCC stem cells (tumour-initiating cells, TIC) [42]. In addition, TF/factor VIIa/PAR_2_-signalling cascade seems to be involved in the modulatory mechanisms of m-TOR-mediated autophagy in HCC [43]. Overall, these data suggest a role for PAR_2_ in HCC progression.

At present no information is available about the impact of PAR_2_ in stromal cells for HCC development and progression. However, human HSCs have been shown to express PAR_2_ and upon its activation increase the release of collagen [44] and augment inflammatory and pro-fibrotic pathways through the induction of pro-inflammatory cytokines and extracellular matrix proteins [45]. This indicates that PAR_2_ stimulates the activation, proliferation, collagen production, and TGF-β protein production by HSCs [45]. Therefore, we specifically addressed this issue by evaluating the effect of PAR_2_ silencing in the HSC cell line LX-2 [46] on tumour growth in a *SCID* mouse xenograft model, in which a HCC was induced by (co)injection of LX-2 cells and Hep3B liver carcinoma cells.

## Results

### PAR_2_ knockdown inhibits tumour growth in a HCC-*SCID* mouse model

**Fig. 1:**
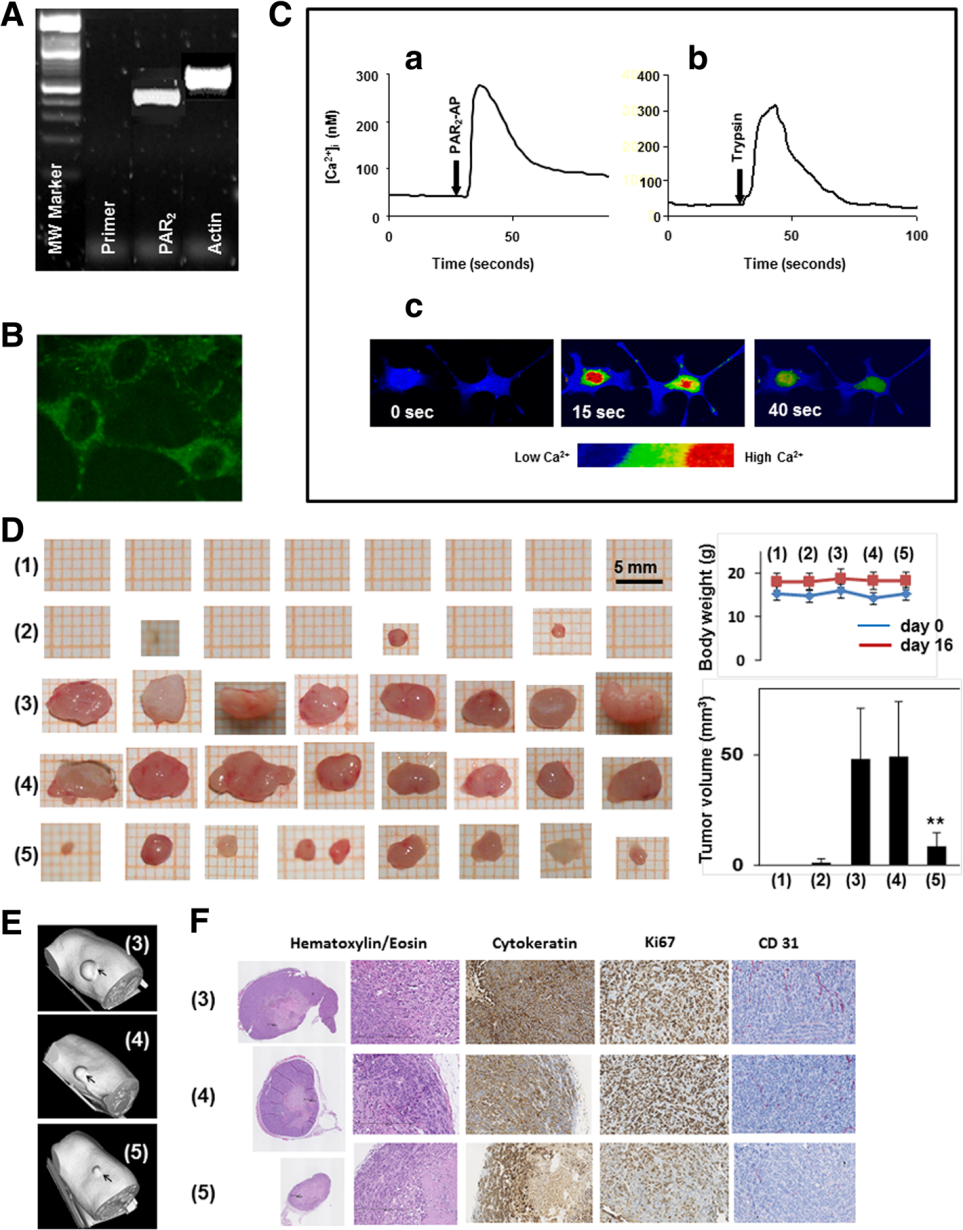
PAR_2_ knockdown in LX-2 cells inhibits tumour growth in a *SCID* mouse model. **a**-**c** Expression and function of PAR_2_ in LX-2 cells. **a** RT-PCR of PAR_2_ expression. Extraction of total RNA from the LX-2-wt cells and synthesis of cDNA was performed as described in the Methods section. PCR reactions without cDNAs were run as a negative control (Primer). Integrity of the cDNA was independently confirmed by amplification of beta-actin (Actin). MW marker, molecular-weight marker. Representative results of three independent experiments are shown. **b** PAR_2_ immunofluorescence was detected using the confocal laser scanning microscope LSM-510 Meta (Carl Zeiss, Germany). Localization of immunofluorescence labelled PAR_2_ is shown in permeabilized LX-2-wt cells using SAM-11 (1:100) and a FITC-conjugated anti-mouse IgG (1:200) as secondary antibody. **c** LX-2-wt cells grown on Lab Tek chambered borosilicate cover glass were loaded with fluo-4-AM as described in Methods. For calcium measurements, an inverted confocal laser scanning microscope LSM 510 was used. Fluorescence was monitored at 488 nm. **(a)** PAR_2_-AP (10 µM) and **(b)** trypsin (10 nM) induce Ca^2+^ rise in LX-2 cells. **(c)** Fluorescence images, in pseudocolor, from single LX-2 cells. The sequence shows a fast and transient fluorescence increase from 15 s to 40 s after PAR_2_-AP addition (0 s). Data represent the mean ± SD from calcium measurements in 20 individual cells, respectively. (**d**-**f**) PAR_2_ knockdown in LX-2 cells inhibits tumour growth in a *SCID* mouse model. *SCID* mice were randomized into five groups, each consisting of 8 animals. Hep3B and LX-2 cells were subcutaneously (co)injected at the right flank of the mice [(1): 5 × 10^5^ LX-2-wt; (2): 10^5^ Hep3B cells; (3): 10^5^ Hep3B cells plus 5 × 10^5^ LX-2-wt, (4): 10^5^ Hep3B cells plus 5 × 10^5^ LX-2-shCo cells, (5): 10^5^ Hep3B cells plus 5 × 10^5^ LX-2-shPAR_2_ cells]. **d** Tumours prepared 16 days after xenotransplantation. Data for body weight and tumour volume are indicated as mean ± SD (*n* = 8/group); **p < 0.01). **e** Representative micro CT images from the tumours 16 days after xenotransplantation. For 3D reconstruction of the tumours the software “Imalytics” (Philips GmbH, Aachen, Germany) was used. **f** Paraffin sections obtained from tumours and stained for hematoxylin/eosin, cytokeratin, Ki67 or CD31. Images were taken at magnification = × 5 (*left panel*) and at magnification = × 200

Activated HSCs are known to promote HCC growth and progression [7–18], however, whether HSC-expressed PAR_2_ is involved here remains unclear. To analyse this, we employed the human HSC cell line LX-2 in subcutaneous tumourigenicity experiments in a HCC-*SCID* mouse model. Although PAR_2_ expression by HSCs has been reported [44, 45], specific data for LX-2 cells in this regard were not available. PAR_2_ expression was therefore analysed by PAR_2_-specific reverse transcription-polymerase chain reaction (RT-PCR), confocal immunofluorescence and electron microscopy. Expression was readily detected at both the mRNA (Fig. 1a) and protein level (Fig. 1b). Granular PAR_2_ immunoreactivity was prominently visible around the nucleus, and to a lesser extent in the peripheral cytoplasm and the membrane compartment (Fig. 1b). Membrane localization of PAR_2_ was also found using scanning electron microscopy techniques and immunogold labeling (Additional file 1: Figure S1). To verify that the PAR_2_ protein on LX-2 cells is signalling-competent, [Ca^2+^]_i_ mobilisation in response to ligand stimulation was used as an index for PAR_2_ activation [47]. We observed a strong effect of both the synthetic PAR_2_-AP, 2-furoyl-LIGRLO-NH_2_(10 µM), and trypsin (10 nM) on free intracellular calcium (Fig. 1c). The concentration dependency and data for PAR_2_ specificity of [Ca^2+^]_i_ mobilisation induced by PAR_2_-AP are shown in Additional file 2: Figure S2.

**Fig. 2:**
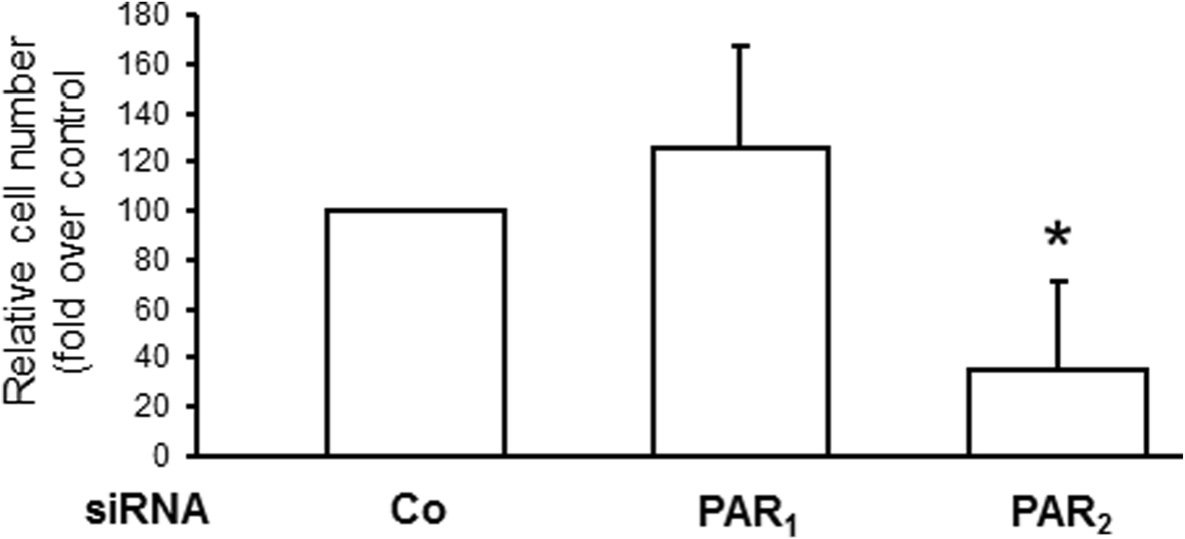
PAR_2_ knockdown inhibits the pro-mitotic effect of LX-2 cell culture supernatants on Hep3B cells. LX-2 cells were treated on two consecutive days with transfection agent (Lipofectamine 2000) alone (-) or Lipofectamine 2000 plus 50 nM each of a universal control siRNA (Co) or siRNA to PAR_1_ or PAR_2_. After the second transfection, cells received normal growth medium which remained on the cells for 48 h. The resulting culture supernatants from PAR_2_ or PAR_1_ depleted and control LX-2 cells were then harvested, cleared by centrifugation and transferred to cultures of Hep3B cells. After a 72 h incubation period with the LX-2 conditioned media, Hep3B cells were detached and cell numbers determined by manual counting and trypan blue exclusion test. Data represent relative cell numbers (mean ± S.D.) from three independent experiments with cell numbers of Co cells set at 100 %. * *p* < 0.05 versus control

**Fig. 3:**
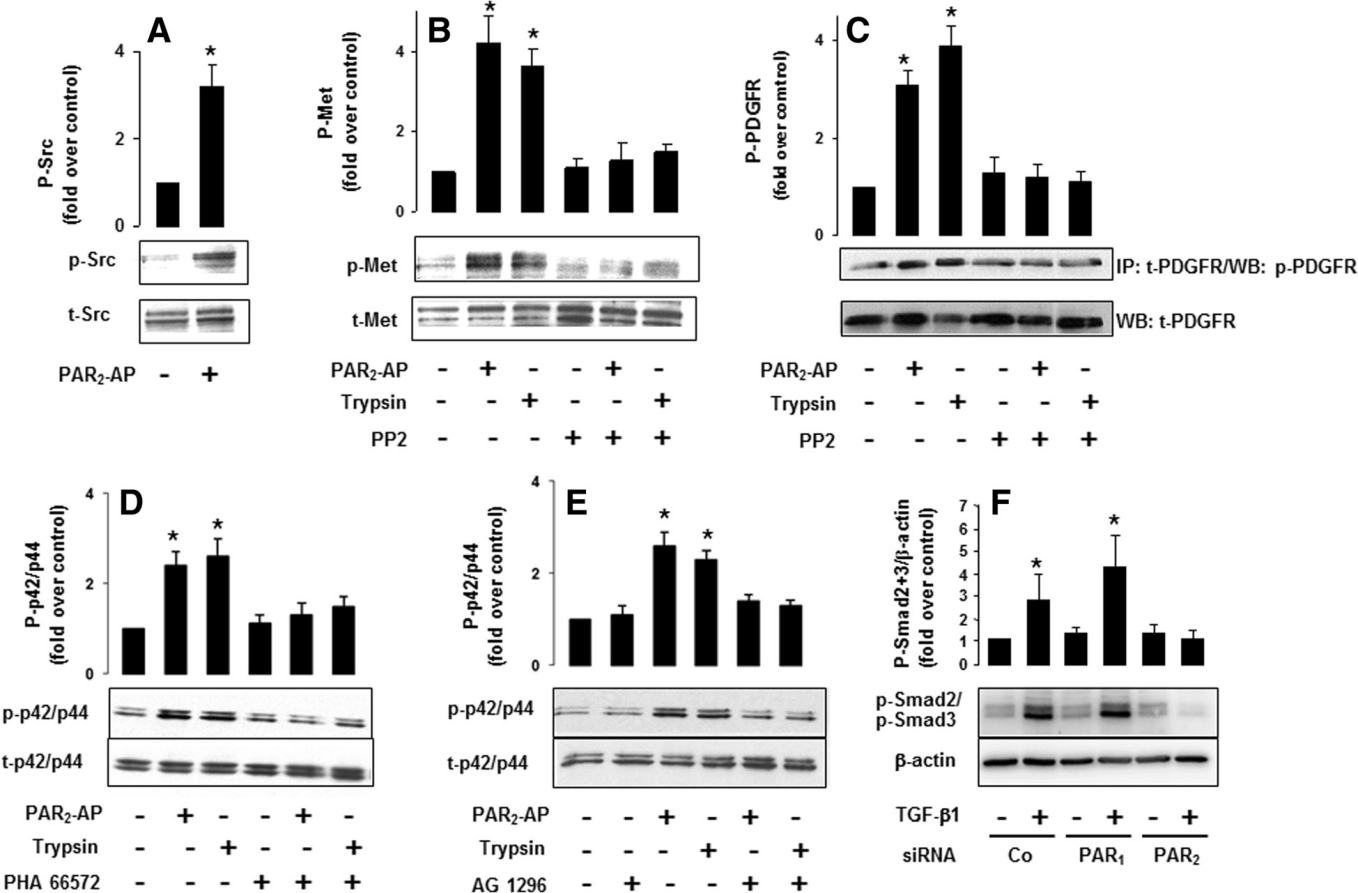
PAR_2_ mediates activation of Src, Met, PDGFR and p42/p44 MAPK. LX-2-wt cells were cultured serum-starved for 17 h. **a** The cells were treated with PAR_2_-AP (10 μM) for 3 min and cell lysates were subjected to SDS-PAGE and Western blotting with an anti-phospho-Src antibody and re-probed with an anti-Src antibody. P-Src = phosphorylated Src, t-Src = total Src. **b** The cells were treated with PAR_2_-AP (10 μM) or (**b**-**e**) trypsin (10 nM) for 3 min and cell lysates were subjected to SDS-PAGE and Western blotting with an anti-phospho-Met antibody and re-probed with an anti-Met antibody. P-Met = phosphorylated Met, t-Met = total Met. **c** The cells were treated with PAR_2_-AP (10 μM) or trypsin (10 nM) for 3 min and cell lysates were subjected to SDS-PAGE and Western blotting with an anti-phospho-PDGFR antibody and re-probed with an anti-PDGFR antibody. P-PDGFR = phosphorylated PDGFR, t-PDGFR = total PDGFR. Serum-starved LX-2-wt cells were preincubated with **d** vehicle or the Met receptor tyrosine kinase inhibitor, PHA 665752 (0.1 μM) for 1 h or **e** with vehicle or the PDGFR inhibitor AG 1296 (1.0 μM) for 1 h. Following stimulation for 10 min with the respective receptor agonist cell lysates were immunoblotted with an anti-phospho-p42/p44 MAPK antibody and re-probed with a p42/p44 MAPK antibody. P-p42/p44 = phosphorylated p42/p44 MAPK, t-p42/p44 = total p42/p44 MAPK. **f** LX-2-wt cells were depleted of PAR_2_ or PAR_1_ protein by siRNA transfection as described in Fig. 2. Forty-eight hours after the second transfection, the growth medium was removed and replaced by fresh medium supplemented or not with 10 ng/ml TGF-β1. After 30 min, cells were lysed and immunoblotted with anti-phospho-Smad2/3 antibody and subsequently with a β-actin antibody to verify equal loading. The phosphorylated forms of Smad2 and Smad3 run as closely spaced bands due to the slightly lower electrophoretic mobility of Smad2. **a-f** Quantification of immunoblots was performed by scanning densitometry (Image J 1.43; National Institutes of Health, Bethesda, Maryland, USA). The data shown in the histograms above the blots are expressed as the fold increase over untreated control (averages ± S.D.) from three independent experiments. *P-value < 0.05 versus non stimulated control

**Fig. 4:**
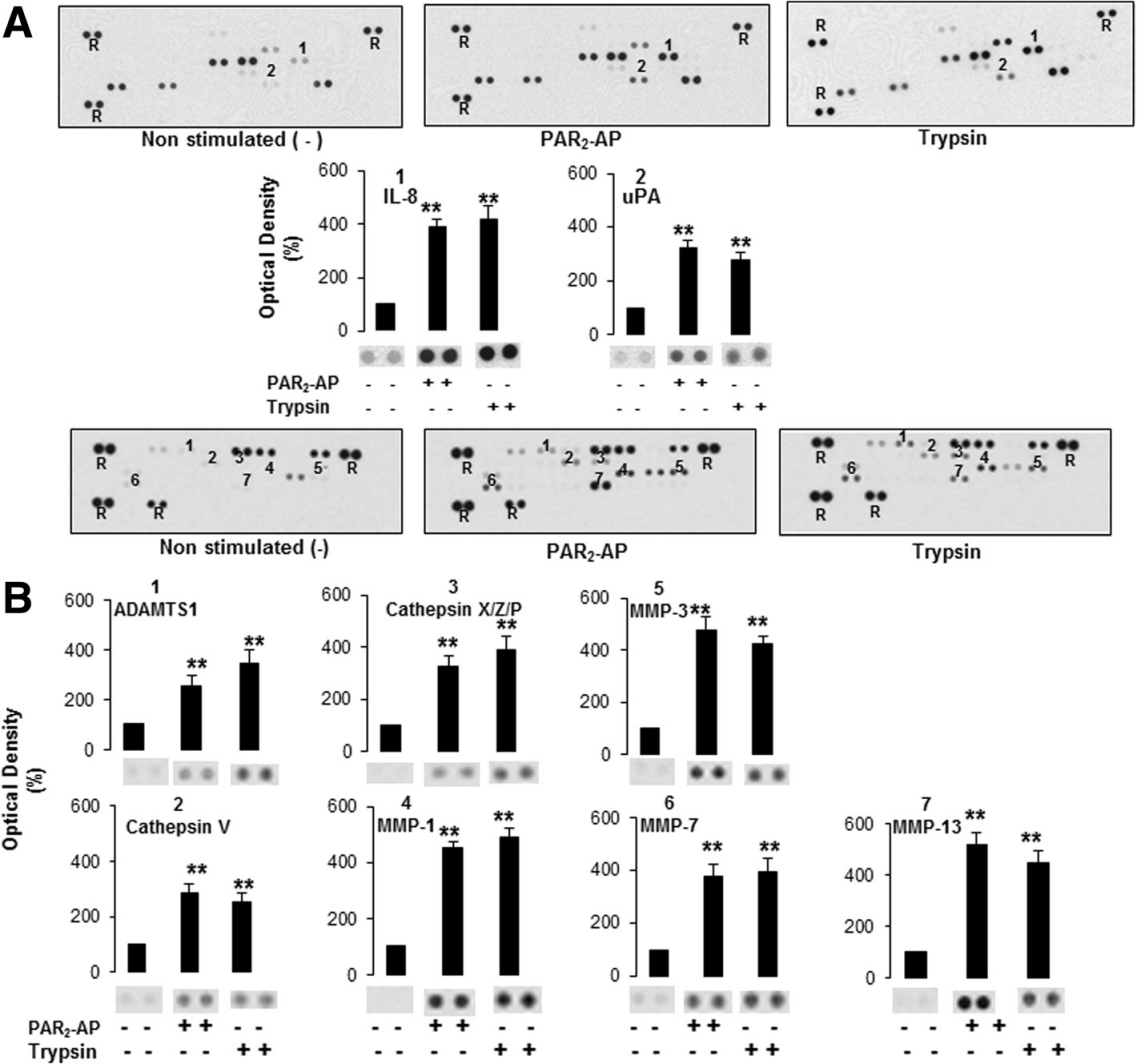
LX-2 cells stimulated with PAR_2_-AP secrete enhanced level of proliferation- and angiogenesis-associated cytokines and proteinases. LX-2-wt cells, serum-starved for 17 h were treated with PAR_2_-AP (10 μM), trypsin (10 nM) or vehicle for 24 h and the supernatants were evaluated by protein analyses using **a** Human Angiogenesis Array Kit and **b** Human Protease Array kit as described in the Method section. In the representative images each pair of horizontal blots (bands) represents a different protein present in the supernatant, whereas the intensities of the blots characterised the amount of the respective protein (R = reference spot for protein loading). The results in the histograms below the images represent the mean ± SD for three independent experiments [**indicates a significant difference from non-stimulated (*p* < 0.05)]

**Fig. 5:**
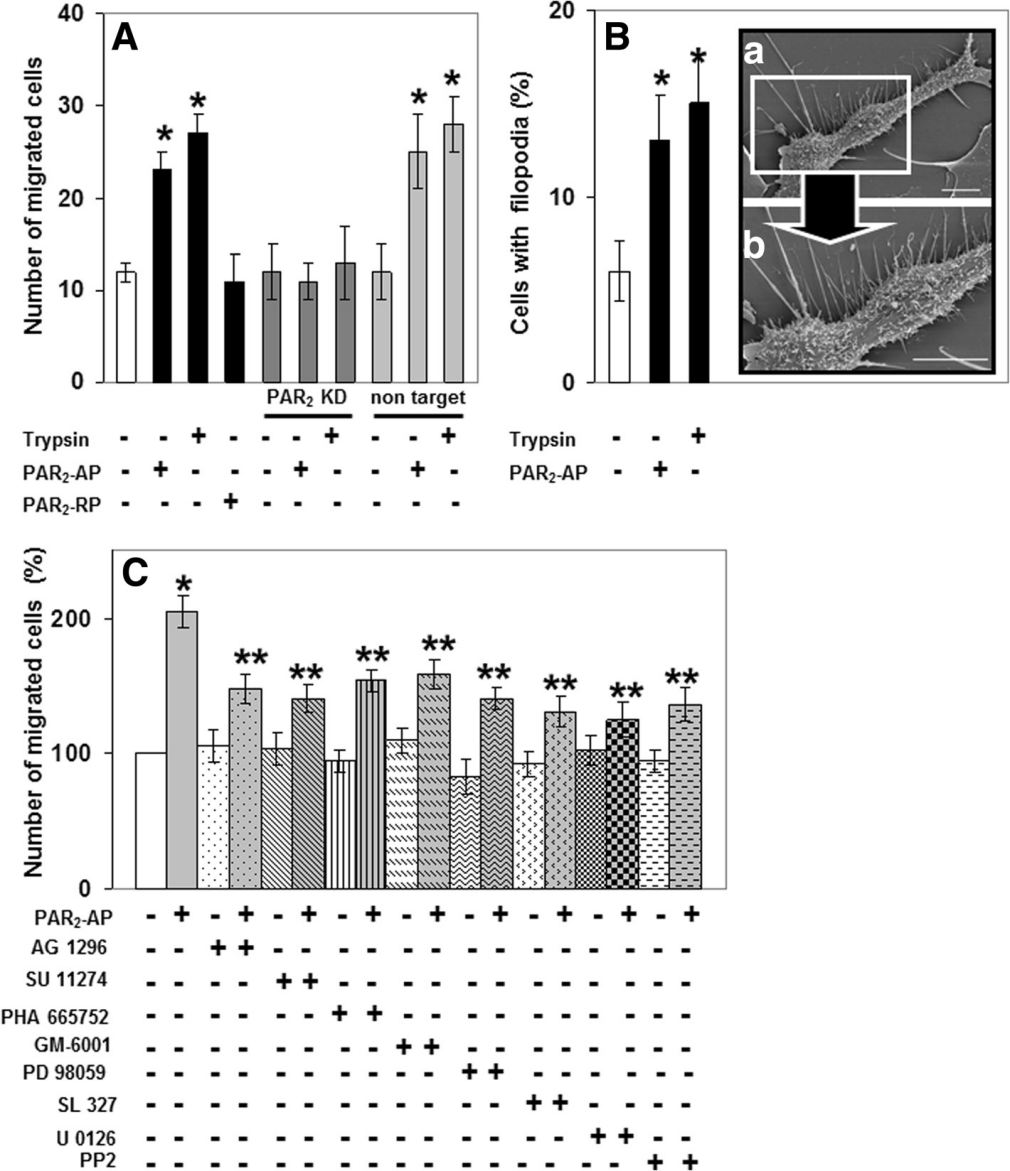
PAR_2_ activation mediates enhanced migration of LX-2 cells in a Met-, PDGFR-, Src- kinase-, p42/p44 MAPK- and MMP-dependent manner. **a** LX-2 cells (wt, shPAR_2_ or shCo as indicated) were serum-starved for 17 h and treated with PAR_2_-AP (10 μM), or trypsin (10 nM) for 6 h. Cells had migrated through the collagen barrier and the pores of the polycarbonate membrane were fixed, stained and quantified by microscopic counting. *Bars* represent the mean values ± S.D. of octuplicates obtained in one experiment, which is representative for three independent assays. **P-value < 0.05 versus non stimulated control. **b** Serum-starved LX-2-wt cells were stimulated with PAR_2_-AP (10 μM) for 4 h and analysis of plasma membrane filopodial structures was performed using scanning electron microscopy as described in the method section. (**a**) Cells with filopodia were quantified in a blinded manner from 100 individual cells per each group and from three experimental preparations. (**b**) enlarged inset. The picture shows a single LX-2-wt cell with typical filopodial spike pattern after stimulation with PAR_2_-AP. **c** Serum-starved LX-2-wt cells were preincubated for 1 h with vehicle, SU 11274 (10 μM), PHA 665752 (0.1 μM), AG 1296 (1.0 μM), SL 372 (5.0 μM), PD 98059 (10 μM), U 0126 (10 μM), PP2 (5.0 μM), or GM-6001 (1.0 μM). Cell migration in response to PAR_2_-AP (10 μM) was analysed after 6 h as described under **a**. Representative results from three independent experiments are shown. *P-value < 0.05 versus non stimulated control, **P-value < 0.05 versus stimulated with PAR_2_-AP and versus non stimulated control

Having demonstrated both PAR_2_ expression and function, we went on to study the effect of PAR_2_ knockdown in LX-2 cells on the growth of tumour xenografts in vivo. For that purpose, suspensions of cells from the HCC cell line Hep3B and LX-2 cells (LX-2-wt) stably expressing a short hairpin (sh) RNA directed against PAR_2_ (LX-2-shPAR_2_) and LX-2 cells with a non-target control shRNA, (LX-2-shCo) were injected into the right flank of *SCID* mice. After 16 days, tumour formation was evaluated by macroscopic inspection, micro CT analysis and histochemical/immunohistochemical staining. While injection of LX-2-wt cells did not result in tumour development [Fig. 1d, (1)] and Hep3B cell injection alone yielded only small tumours (tumour volume < 3 mm^3^) in 2 out of 8 mice [Fig. 1d, (2)], simultaneous injection of Hep3B and LX-2-wt cells induced the development of large tumours (tumour volume approximately 50 mm^3^) [Fig. 1d, (3)]. Notably, the tumour promoting effect of LX-2 cells was significantly reduced when LX-2-shPAR_2_ cells were coinjected [Fig. 1d, (5)] but not with LX-2-shCo cells [Fig. 1d, (4)]. In Fig. 1e, representative micro CT images are shown for the tumours that developed under the skin of mice treated with Hep3B and LX-2-wt cells (3) and with simultaneous injection of Hep3B and LX-2-shCo cells (4) or LX-2-shPAR_2_ cells (5). Histochemical staining of tumour sections from experimental groups (2)-(5) by hematoxylin/eosin showed no gross differences and revealed histological features of a HCC with a trabecular growth pattern, central necrosis and pronounced inflammatory component (Fig. 1f). The epithelial nature of the tumours was confirmed by cytokeratin staining with the pan-keratin antibody MNF 116 [48].

To further characterise the inhibitory effect of *F2RL1* silencing in LX-2 cells on tumour growth, immunohistochemical stainings of Ki67 and CD31 were performed to analyse tumour cell proliferation and tumour vascularization, respectively, both being hallmarks of cancer progression and tumour growth [49]. As demonstrated in Fig. 1f, the number of Ki67-positive cells (Hep3B and LX-2 cells) was apparently higher in tumours induced by coinjection of Hep3B cells with LX-2-wt cells (or LX-2-shCo) than with PAR_2_ deficient LX-2 cells (quantification by manual counting revealed approximately 2.5-fold higher number of Ki67-positive cells, data not shown), strongly suggesting that the reduced tumour growth mediated by silencing *F2RL1* in LX-2 cells may depend on diminished cell proliferation in the tumour microenvironment. Next, we assessed whether the reduced tumour growth with LX-2-shPAR_2_ cells correlated with changes in angiogenesis. Therefore, expression of endothelial marker CD31 in sections from tumours induced by coinjection of Hep3B cells with either wt, shCo or shPAR_2_ LX-2 cells were compared. As shown in Fig. 1f, PAR_2_ knockdown in LX-2 cells resulted in remarkably reduced immunostaining for CD31 indicating that LX-2-expressed PAR_2_ promoted angiogenesis. Overall these data suggest that PAR_2_ expression in HSCs promotes HCC growth by increasing cell proliferation and inducing angiogenesis.

### PAR_2_ knockdown inhibits the pro-mitotic effect of LX-2 cell culture supernatants on Hep3B cells

Having shown the inhibitory effect of *F2RL1* silencing in LX-2 cells on tumour growth in vivo, we sought to reveal whether reduced proliferation and/or increased apoptosis of the tumor cells in presence of PAR2-deficient LX-2 cells may be the underlying mechanism. For this purpose, we stimulated Hep3B cells with culture supernatants (conditioned for 48 h) from PAR_2_ depleted LX-2 cells or PAR_1_ depleted LX-2 cells as control. After incubation of Hep3B cells with conditioned media from the various LX-2 transfectants for 72 h, vital cell numbers were dramatically lower in Hep3B cultures which had received conditioned medium from PAR_2_ depleted LX-2 cells (Fig. 2). This effect resulted from reduced proliferation rather than enhanced apoptosis since the number of non-viable cells, as determined by trypan blue exclusion, was not different between the various cultures (data not shown). These data show that PAR_2_ but not PAR_1_ expressed by LX-2 cells is critically involved in stimulating growth of Hep3B cells and attest to the above assumption that reduced tumour growth following *F2RL1* silencing in LX-2 cells depends at least in part on diminished tumour cell proliferation.

### PAR_2_ mediates activation of Src, Met, PDGFR and p42/44 MAPK and promotes TGF-β-induced Smad activation

Based on the results in vivo demonstrating that PAR_2_ knockdown in LX-2 cells resulted in remarkably reduced tumour growth in a xenograft *SCID* mouse model, we set out to analyse in more detail how PAR_2_ impacts on LX-2 cells. We first focussed on signalling pathways known to be triggered by PAR_2_ activation [24]. PAR_2_-mediated activation of different intracellular effectors was monitored by immunoblotting. Stimulation of LX-2 cells with the PAR_2_ agonists trypsin (10 nM) or PAR_2_-AP (10 μM) caused a marked increase of phosphorylated (p)-Src (Fig. 3a), p-Met (Fig. 3b), p-PDGFR (Fig. 3c) and p-p42/p44 MAPK immunoreactivity (Fig. 3d and e). To define the PAR_2_-initiated phosphorylation sequence, we performed different inhibition experiments. We found in LX-2 cells that trypsin and PAR_2_-AP-induced phosphorylation of Met and PDGFR could be blocked by PP2 (Fig. 3b and c), while the Met inhibitor PHA 665752 and the PDGFR antagonist AG 1296 were able to inhibit PAR_2_-mediated phosphorylation of p42/p44 MAPK (Fig. 3d and e).

TGF-β is overexpressed in HCC [50, 51] and considered to be a major factor promoting liver carcinogenesis. Specifically, TGF-β drives transformation of HSCs into myofibroblasts [52, 53] as well as their migration and invasion [54]. PAR_2_ may enhance these pro-oncogenic effects by stimulating TGF-β gene expression and protein production in HSCs [45]. However, based on recent data from our group [55], PAR_2_ may also promote TGF-β signaling in a more direct fashion in HSCs/LX-2 cells. Indeed, small interfering RNA (siRNA)-mediated depletion of PAR_2_ in LX-2 cells impaired the ability of TGF-β1 to induce C-terminal phosphorylation of Smad2 and Smad3, reflecting activation of the pathway, in response to TGF-β1 challenge (Fig. 3f).

Taken together, these data indicate that PAR_2_ activation stimulates a Src-Met/PDGFR-p42/p44 MAPK signalling axis in LX-2 cells while PAR_2_ protein expression is required for activation of TGF-β/Smad signalling.

### PAR_2_ stimulation in LX-2 cells increases secretion of pro-angiogenic and pro-mitotic factors and proteinases

We next assessed the possibility that PAR_2_ activation in LX-2 cells may result in the secretion of pro-mitotic and pro-angiogenic factors, in turn promoting tumour cell growth. For this purpose proteome profiler arrays were employed allowing for simultaneous detection of 55 cytokines and 35 proteinases. Using these arrays for analysis of culture supernatants of LX-2 cells stimulated with PAR_2_-AP or trypsin for 24 h, or non-stimulated LX-2 cells as control, revealed predominantly enhanced levels of IL-8, uPA (Fig. 4a), cathepsins V, X, Z, P, MMPs 1, 3, 7, 13 and ADAMTS1 (Fig. 4b) in supernatants of LX-2 cells treated with PAR_2_-AP or trypsin, compared to control cells.

### PAR_2_ activation increases migratory capabilities of LX-2 cells in a Src-, Met-, PDGFR-, p42/p44 MAPK- and MMP-dependent manner

HSC locomotion is known to be essential for disease processes in liver including fibrosis and cancer [56, 57]. Specifically, activated HSCs infiltrate HCC stroma and peri-tumoural tissue during hepatocarcinogenesis where they secrete substantial amounts of bioactive proteins [58] thereby facilitating HCC development, progression and metastasis. Since PAR_2_ activation in LX-2 cells led to activation of signalling pathways driving cell motility (see Fig. 3) and to secretion of pro-invasive proteases (see Fig. 4), we addressed the question of whether these events were associated with increased migration of LX-2 cells. Using a boyden-chamber assay, we found that stimulation of LX-2 cells for 6 h with trypsin or PAR_2_-AP, but not with the reverse PAR_2_-AP (2-furoyl-OLRGIL-NH_2_, PAR_2_-RP), significantly enhanced their migration (Fig. 5a). As further shown in Fig. 5a, the effect of trypsin and PAR_2_-AP on cell migration was absent in LX-2-shPAR_2_ cells.

It is well known that a migratory phenotype can be associated with epithelial-mesenchymal transition (EMT), which in turn involves morphological changes, such as lamellipodial protrusions and filopodial spikes [59]. Therefore, scanning electron microscopy analyses on the various LX-2 cells were performed. Interestingly, treatment of LX-2 cells with PAR_2_-AP or trypsin induced the formation of filopodia spikes in a significantly larger number of cells when compared with non-stimulated controls (Fig. 5b).

To assess the contribution of the PAR_2_ activated signalling pathways (see Fig. 2) for LX-2 cell migration, we again performed inhibition studies with a battery of protein tyrosine kinase and MMP inhibitors. As shown in Fig. 5c, PP2, PHA 665752 and SU 11274 (another Met inhibitor), AG 1296, the MEK inhibitors PD 98059, SL 327 and U 0126, and the MMP inhibitor GM-6001 all inhibited the effect of PAR_2_-AP on migration of LX-2 cells. Overall, these data support the view that PAR_2_ activation increases the invasive capabilities of LX-2 cells via activation of Src, PDGFR, Met, p42/p44 MAPK and MMPs.

## Discussion

During the last years it became evident that PAR_2_ plays an important role in the development, progression and metastasis of tumours especially from epithelial origin [30–39, 60]. In case of HCC, studies have focussed on the function of PAR_2_ expressed by the tumour cells. This PAR-subtype is known to promote migration and invasion of HCC cells by stimulating various intracellular signalling systems including calcium mobilisation, reactive oxygen species, Met and p42/p44 MAPK [40, 41]. Moreover, PAR_2_ is involved in the regulation of CD47^+^ HCC stem cells, contributing to tumour initiation, self-renewal and metastasis [42]. In addition, it could be shown that the TF/factor VIIa/PAR_2_-signalling cascade stimulates m-TOR signalling in liver carcinoma cells and reduces m-TOR-mediated autophagy induction in HepG2 xenografts in a *SCID* mouse model, suggesting a role in HCC progression [43].

Recent data indicated that also the stromal compartment plays a crucial role in HCC development and progression and provides an attractive target for HCC therapy [11, 14]. In the liver, HSCs, fibroblasts, myofibroblasts as well as immune and endothelial cells represent the major cell types of the hepatocyte stromal microenvironment. Among them, activated HSCs, beside their well-studied role in liver fibrosis and extracellular matrix remodeling [61], are discussed as a key driver for liver cancer development and progression [7–18]. HSC activation is regulated by complicated networks of numerous growth factors, proteinases, cytokines, integrins and their cognate receptors including the PAR family receptors [61, 62]. PAR_2_ has been shown to stimulate activation, proliferation, collagen synthesis and TGF-β production in HSCs and to promote experimental liver fibrosis [45, 63–65].

In this study, we provide for the first time evidence that PAR_2_ in HSCs stimulates HCC growth. This was concluded from the finding that *F2RL1* knockdown in hepatic stellate LX-2 cells inhibited the growth-promoting effect of these cells in a HCC xenograft *SCID* mouse model, as demonstrated by significantly smaller volumes of the tumours established in mice by coinjection of Hep3B and LX-2 cells depleted of PAR_2_. In addition, immunohistochemical analyses of these tumours revealed lower indices for proliferation (reduced immunoreactivity of Ki67 in both Hep3B and LX-2 cells), and angiogenesis (reduced CD31 in endothelial cells). This suggests a promoting role for HSC-derived PAR_2_ in HCC growth through pro-proliferative, pro-angiogenic and possibly fibrotic effects on tumour and stromal cells in the HCC microenvironment. The pro-mitotic effect of HSC-derived PAR_2_ was subsequently shown in cell counting experiments with Hep3B cells stimulated with conditioned media from PAR_2_ depleted LX-2 cells. Of note, this growth promoting effect was specific to PAR_2_ and was not observed for the related PAR_1_. These data are in agreement with the in vivo data and show that HSCs act on tumour cells through the release of soluble mitogenic factors. We also observed that transfection of PAR_2_ siRNA decreased LX-2 cell numbers (data not shown), suggesting the possibility that HSC-derived PAR_2_ also contributes to tumour growth by promoting HSC proliferation in a cell-autonomous or auto-/paracrine manner. Given that activated HSCs are the major matrix producing cells in the liver, this may secondarily result in enhanced total matrix accumulation and eventually fibrosis.

It is now established that HSCs may facilitate HCC development, progression and metastasis by different processes including migration to the cancer site. More specifically, during cancer development activated HSCs infiltrate HCC stroma and peri-tumoural tissue and secrete substantial amounts of bioactive proteins [58] which may act either directly on the cancer cells or help to establish a microenvironment permissive for HCC progression. Migration of HSCs is known to be driven mainly by PDGF/PDGFR-dependent mechanisms [66–68], including a regulatory role for nitric oxide (NO) and the small GTPases, Rac1 and Rho [69, 70]. Notably, PAR_2_ induced the activation of PDGFR and Met following stimulation with PAR_2_-AP or trypsin, suggesting the possibility that PAR_2_ utilizes these pathways to drive the migratory capacity of LX-2 cells. The importance of this PAR_2_-PDGFR-Met crosstalk was further supported by the finding that the PAR_2_-mediated migration of LX-2 cells was blocked by AG 1296 and SU 11274/PHA 665752, respectively. In addition, we provided evidence for a role of Src and p42/p44 MAPK in PAR_2_-PDGFR/Met-induced signalling to PAR_2_-induced invasion in LX-2 cells, since this could be inhibited by PP2 and SL 327/U 0126, respectively, and stimulation of PAR_2_ with PAR_2_-AP or trypsin induced phosphorylation of Src and p42/p44 MAPK. Moreover, the PAR_2_-induced effect on p42/p44 MAPK could be blocked by AG 1296 and PHA 665752. These results also demonstrate that the PAR_2_-triggered phosphorylation of Src is upstream while p42/p44 MAPK is downstream of PDGFR and Met activation in LX-2 cells. Our results further indicate that, in addition to a Src-PDGFR/Met-p42/p44 MAPK signalling axis, MMP activity seems to be involved in PAR_2_-mediated invasive effect on LX-2 cells as concluded from the findings that LX-2 cells treated with PAR_2_-AP secrete MMPs 1, 3, 7, 13 and that the MMP inhibitor GM-6001 inhibited the effect of PAR_2_-AP and trypsin on LX-2 cell migration.

PAR_2_ activation augments inflammatory and pro-fibrotic pathways through the induction of genes encoding pro-inflammatory cytokines and extracellular matrix proteins [45]. In line with this, our in vitro experiments with LX-2 cells showed that activated PAR_2_ not only stimulated cell migration but also the release of IL-8, uPA, cathepsins, MMPs and ADAMTS 1. All these factors are known to contribute to HCC initiation, growth and progression of HCC by degrading and remodeling the extracellular matrix and by stimulating angiogenesis and immunomodulation [71–77].

In addition to pro-inflammatory cytokines, TGF-β is considered a major factor promoting liver carcinogenesis. TGF-β1 and TGF-β2 are overexpressed in HCC [50, 51] and may also drive transformation of HSCs into myofibroblasts [52, 53] as well as their migration and invasion [54]. Notably, HSCs and LX-2 cells are sensitive to TGF-β treatment [46, 61], and TGF-β gene expression and protein production in HSCs is stimulated by PAR_2_ [45]. Prompted by recent observations in other cell types [55], we hypothesised that PAR_2_ expression is also required for TGF-β signaling in a cell-autonomous manner. To this end, depletion of PAR_2_ but not PAR_1_ from LX-2 cells inhibited the ability of TGF-β1 to activate Smad2 and Smad3. This suggests that HSC-derived PAR_2_, in addition to driving tumour cell and HSC proliferation may promote TGF-β sensitivity of HSCs and, as a consequence, matrix production.

Evidence for a crucial role for stromal cell-derived PAR_2_ in promoting primary tumour growth came also from mouse models for other solid and stroma-rich cancer types. PAR_2_ deficient mice (harbouring PAR_2_ deficient stromal cells) exhibit decreased tumour growth and metastasis in a spontaneous polyoma middle T (PyMT) breast cancer model [78]. In a mouse model of pancreatic cancer, stromal PAR_2_ promoted primary tumour growth [79]. In tumours resulting from B16 melanoma cells injected subcutaneously, PAR_2_ limited primary tumour growth [80]. The role of stromal PAR_2_ in metastasis, however, appears to be strongly context-dependent. Stromal PAR_2_ limited lymph node metastasis in a spontaneous polyoma middle T (PyMT) breast cancer model [78] and in the pancreatic cancer mouse model [79] but enhanced spontaneous metastasis of B16 melanoma cells injected subcutaneously [80]. Finally, PAR_2_ has been shown to play a critical role in stimulating tumour angiogenesis in the context of mammary carcinoma [81].

## Conclusion

Our study provides novel insight into the function of PAR_2_ in HSCs, which may be involved in HCC growth and progression. PAR_2_ induces secretion of pro-angiogenic and pro-mitotic factors and proteinases, directly stimulates tumour cell growth in vitro through secreted factors and activates various signalling pathways (Src, Met, PDGFR, p42/p44 MAPK) being related to HSC growth and matrix production. It can be hypothesised that these mechanisms are of importance in the HCC microenvironment where both tumoural and stromal cells may produce PAR_2_-activating proteinases, like the kallikrein-related peptidases [82, 83] driving PAR_2_-mediated effects in HSCs on HCC growth.

Numerous studies convincingly demonstrate that HSCs play a critical role in HCC growth and progression [7–18]. From our data on LX-2 cells PAR_2_ may be concluded as a player in this scenario. This was supported by preliminary data demonstrating PAR_2_ function also in primary cultures of human HSCs (R.K. and H.U., unpublished observation). Since we used exclusively Hep3B cells as a HCC cellular model so far, analogous studies with other HCC cell lines and well-characterised primary HCC cultures are warranted to confirm the tumour-promoting function of PAR_2_ in HSCs.

Our data suggest that targeting PAR_2_ in HSCs may have potential for the treatment of this liver carcinoma.

## Methods

### Reagents

Trypsin (EC 3.4.21.4; 14,700 U/mg) was obtained from Sigma-Aldrich Chemie GmbH (Steinheim, Germany), and the selective Met receptor tyrosine kinase inhibitors PHA 665752 and SU 11274, the PDGFR inhibitor, tyrphostin AG 1296, the MAPK (MEK) inhibitors, PD 98059, U0126 and SL 327, the MMP inhibitor GM-6001 (galardin) and the Src inhibitor PP2 were from Calbiochem/Merck Biosciences (Bad Soden, Germany). Trypan blue exclusion tests confirmed that these inhibitors did not impair cell viability of LX-2 cells (approx. 95 % viability after treatment for 24 h, data not shown). Rabbit polyclonal anti-phospho-Met-antibody [pYpYpY1230/1234/1235] and rabbit polyclonal anti-phospho-PDGFR-α [Tyr849]/PDGFR-β [Tyr857] were from Life Technologies GmbH (Darmstadt, Germany), a phosphospecific monoclonal antibody to p42/p44 MAPK, and rabbit polyclonal anti-PDGFR-α antibody, C-20, a polyclonal anti-p42/p44 MAPK antibody, a polyclonal anti- Met (C12) antibody and a mouse monoclonal anti-PAR_2_ antibody, SAM 11, were from Santa Cruz Biotechnology, Inc., Heidelberg, Germany. A polyclonal rabbit anti-phospho-Smad2 (S465/S467)/Smad3 (S423/S425) antibody (#AB3226) was from R&D Systems (Wiesbaden, Germany) and an anti-β-actin antibody from Sigma. A rabbit polyclonal anti-Src [pY418] antibody was from BioSource Europe, S.A., Belgium and a monoclonal anti-Src antibody was from Upstate Biotechnology, Lake Placid, NY, USA. As secondary antibodies goat anti rabbit IgG-HRP-conjugated, goat anti mouse IgG-HRP-conjugated and goat anti rabbit IgG-FITC conjugated (Santa Cruz Biotechnology, Inc., Heidelberg, Germany) were used. The PAR_2_ antagonist GB 88 (5-isoxazoyl-Cha-Ile-spiroindene-1,4-piperidine), which selectively blocks PAR_2_ calcium signalling [84] was a kind gift from Dr. David Fairlie, (Institute for Molecular Biosciences, University of Adelaide, Australia).

### Peptide synthesis

The PAR_1_-selective peptide, TFLLRN-NH_2_, PAR_2_-AP, 2-furoyl-LIGRLO-NH_2_ and PAR_2_-RP 2-furoyl-OLRGIL-NH_2_, were synthesised by Fmoc strategy on an ABI-Peptide-Synthesizer 433A using H-Rink Amide ChemMatrix®resin (capacity 0.47 respectively 0.52 mmol/g; PCAS BioMatrix Inc, Canada). The furoyl-group was coupled manually with 5 equivalents of acid. The cleavage of the peptides from resin was performed with trifluoroacetic acid, 5.0 % H_2_O und 3.0 % triisopropylsilane. The peptides were precipitated by diethylether and lyophilized. Crude synthetic peptides were purified using preparative HPLC on a 30 × 250 mm Kromasil C18-column with a flow rate of 40 ml per minute under standard conditions (buffer A: 0.2 % TFA in water, buffer B: 0.2 % TFA in water:acetonitrile, 1:4). The purified peptides were dried by lyophilization and characterised by analytical HPLC and mass spec analysis on a Voyager-DE PRO workstation.

### Cell culture

LX-2 cells (gift from Prof. Scott Friedman, Mount Sinai School of Medicine, New York, USA) were cultured in Dulbeccos modified Eagles’s medium (DMEM) supplemented with 2.0 % fetal calf serum at 37 °C in a humidified atmosphere of 5.0 % CO_2_. The medium was changed every 2–3 days. Hep3B HCC cells (German Collection of Microorganisms and Cell Cultures, Braunschweig, Germany, ACC 325) were routinely cultured in RPMI-1640 supplemented with 10 % fetal calf serum at 37 °C in a humidified atmosphere of 5.0 % CO_2_. The medium was changed every 2–3 days and for subculturing cells were trypsinised. Since trypsin itself activates PAR_2_ the subcultured cells were re-fed sufficiently to remove all traces of trypsin.

### [Ca^2+^]_i_ measurements

[Ca^2+^]_i_ was measured in single cells with fluo-4, a fluorescence indicator for free Ca^2+^. Cells were grown on Lab-Tek™ chambered borosilicate cover glass (Nunc GmbH & Co. KG, Wiesbaden, Germany) and washed twice with HEPES buffer (pH 7.4) containing 10 mM HEPES, 145 mM NaCl, 0.5 mM Na_2_HPO_4_, 6.0 mM glucose, 1.0 mM MgSO_4_, 1.5 mM CaCl_2_. Cells were incubated for 15 min at 37 °C in the same buffer containing 0.5 μM fluo-4 acetoxymethylester (fluo-4-AM). After loading, the cells were washed twice and reincubated in HEPES buffer. For calcium measurement in single cells, an inverted confocal laser scanning microscope (LSM 510, Carl Zeiss, Göttingen, Germany) was used. Fluorescence images were collected by using the 488 nm argon ion laser line and a 505 nm long pass filter. All fluorescence measurements were made from subconfluent areas of the dishes, enabling the ready identification of individual cells. Image data were analysed using the Carl Zeiss AIM software Version 4.2. A selected image in each image set was used as a template for designating each cell as a region of interest.

The intracellular calcium concentration was calculated using the equation [Ca^2+^]_i_ = 345 (F-F_min_)/(F_max_-F) [85]. The Ca^2+^ affinity of fluo-4 (Kd) is 345 nM [86]. F_max_ was obtained by addition of 10 μM ionomycin (+6.0 mM CaCl_2_), F_min_ by addition of 10 mM ethylene glycol-bis(2-aminoethylether)-N,N,N’,N’-tetraacetic acid (EGTA). The average of calcium data obtained by measuring 20 cells was used for calibration.

### Reverse Transcription (RT)-PCR analysis

Total RNA was extracted from 1 × 10^7^ LX-2 cells (RNeasy ® Mini Kit, Qiagen GmbH, Hilden, Germany), and for RT-PCR analysis the Reverse Transcription System (Cat. No. A 3500) from Promega Corporation (Madison, WI, USA) was used. For PCR amplification the following primer pairs were used: forward primer: 5’-TGGATGAGTTTTCTGCATCTGTCC-3’ and 5’-CGTGATGTTCAGGGCAGGAATG-3’. The primers were constructed to generate a fragment of 490 bp for PAR_2_. PCR amplification was performed with Taq polymerase for 32 cycles at 94 °C for 30 s, 55 °C for 30 s, and 72 °C for 30 s, and, finally 72 °C for 5 min [87]. Amplified samples were electrophoresed on a 2.0 % agarose gel containing 0.2 μg/ml SybrGreen and visualized under UV transillumination. In addition, PCR products for PAR_2_ of LX-2 cells were purified and DNA sequences were determined by 4base lab GmbH, Reutlingen (Germany).

### Transient transfection of LX-2 cells with PAR_2_ siRNA and TGF-β stimulation

For transient silencing of *F2RL1*, LX-2 cells seeded on day 1 (2.8 × 10^5^/6-well) underwent two rounds of a 4-h transfection on days 2 and 3 with 50 nM of siRNA to PAR_2_ (Invitrogen) and Lipofectamine 2000 transfection reagent (Life Technologies). As control, a siRNA to PAR_1_ or a universal control siRNA (both from Life Technologies) was transfected in parallel. Successful knockdown of PAR_2_ and PAR_1_ expression was verified by quantitative real-time (RT)-PCR as outlined elsewhere [55] (data not shown). For sequence information on siRNAs and PCR primers see [55]. Forty-eight hours after the second transfection, supernatants to be used for Hep3B proliferation assays (see below) were collected, centrifuged briefly to remove dead cells and cellular debris and stored frozen until use. The remaining LX-2 cells were stimulated for 0.5 h with TGF-β1 (10 ng/ml, ReliaTech, Wolfenbüttel, Germany) in normal growth medium followed by lysis in RIPA buffer containing protease (Boehringer Complete, PMSF)/phosphatase (NaF, sodium orthovanadate) inhibitors and phosphoimmunoblotting for Smad2/3 (see below).

For testing the effect of PAR_2_ silencing on cell viability, cells were seeded into 24-well plates and after treatment with the respective siRNA the viable cells were counted by trypan blue dye exclusion test.

### Generation of LX-2 cells with stable expression of PAR_2_ small hairpin RNA (shRNA)

Plasmid pLKO.1 vectors encoding shRNA constructs targeting human PAR_2_ and plasmid pLKO.1 encoding a non-targeting control shRNA were obtained from Sigma (MISSION® shRNA lentivirus-mediated transduction system, SHCLNG-NM_005242).

To generate lentiviral particles, HEK293T cells were maintained in DMEM (Biochrom) supplemented with 10 % FCS. The cells were transiently transfected with the pLKO.1-derived plasmids in combination with pRev, pEnv-VSV-G and pMDLg using polyethyleneimine. After 12, 24 and 48 h, media containing the retrovirus particles were collected. LX-2 cells were infected three times with the particles in the presence of 8.0 μg/ml polybrene. Forty-eight hours after transduction, transduced cells were selected with 2.0 μg/ml puromycin. The efficiency of the knockdown was assessed by quantitative real-time RT-PCR and by calcium measurements. PCR amplification was performed with Maxima Hot Start *Taq* DNA Polymerase for 40 cycles at 94 °C for 30 s, 60 °C for 30 s and 72 °C for 30 s and finally 72 °C for 5 min. Primers were forward: 5’-TGGATGAGTTTTCTGCATCTGTCC-3’, and reverse: 5’-CGTGATGTTCAGGGCAGGAATG-3’. The qPCR was performed on a MasterCycler RealPlex4 (Eppendorf, Hamburg, Germany). All values for PAR_2_ expression were normalized to those for the housekeeping gene glyceraldehyde-3-phosphate dehydrogenase (GAPDH) in the same sample.

For calcium measurements the effect of trypsin and the PAR_2_-AP, 2-furoyl-LIGRLO-NH_2_, on [Ca^2+^]_i_ mobilisation in PAR_2_ knockdown-LX-2 cells was investigated as described above.

### Immunofluorescence studies

The expression of PAR_2_ on protein level was established using the monoclonal anti-PAR_2_ antibody SAM-11, generated against a peptide representing amino acids ^37^SLIGKVDGTSHVTG^50^ of human PAR_2_ (Santa Cruz Biotechnology). This antibody has been validated for western blot and immunohistochemical/flow cytometry procedures [88, 89]. LX-2 cells, grown on glass coverslips were washed in TBS containing 4.0 % Tween 20, fixed with 2.0 % paraformaldehyde in 0.1 M cacodylate buffer + 4.0 % Tween 20 and washed with 0,1 M glycine buffer (pH 9.0) as well as with TBS + 4.0 % Tween 20. Thereafter, the cells were treated with 10 mM citrate buffer (pH 6.0) in a microwave oven (Miele Supratronic, M 752) at 80 W, washed in 10 mM Tris/EDTA (1.0 mM) buffer (pH 9.0), followed by rinsing in TBS. Then, cells were covered with the secondary antibody (FITC-conjugated anti-mouse IgG). Control experiments were carried out under omission of the primary anti-PAR_2_ antibody or by using mouse IgG_2a_ instead of SAM-11. LX-2 cells were analysed using the confocal laser scanning microscope LSM 510 Meta (Carl Zeiss). Fluorescence images were collected by using the 488 nm argon ion laser line.

### Field emission scanning electron microscopy (FESEM)

#### PAR_2_ immunolabeling

LX-2 cells grown on glass cover slips were washed three times with PBS, prefixed with 0.1 % glutaraldehyde in PBS and washed four times in PBS. The cells were incubated 30 min in labeling blocking buffer (LBB): 1.0 % BSA, 0.5 % gelatin, 0.005 % Tween-20 in PBS, pH 7.2). For PAR_2_ immunolabeling, monoclonal anti-PAR_2_ antibody, SAM-11, diluted 1:25 in LBB, and as secondary antibody, a goat anti-mouse IgG coupled with 20 nm gold particles (British Biocell International, Cardiff, UK; 1:50) were used. After immunolabeling, the cells were rinsed in PBS three times and fixed with 2.5 % glutaraldehyde in PBS for 30 min. After three washings in PBS the cells were dehydrated in rising ethanol concentrations followed by critical point drying and carbon coating in a BAL-TEC SCD 005 Sputter Coater (BAL-TEC, Balzers, Liechtenstein). The cells were examined in a LEO 1530 Gemini field emission scanning electron microscope (Carl Zeiss) at 5 kV acceleration voltage and a working distance of 7 mm using a Centaurus scintillation type backscatter electron detector (K.E. Developments, UK).

#### Analysis of plasma membrane filopodial structures

LX-2 cells were grown on glass coverslips. After incubation with PAR_2_-AP for 4 h, the cells were fixed with 2.5 % glutaraldehyde and subsequently dehydrated as described for detection of PAR_2_ immunolabeling. The cells were sputter coated with gold and examined in a LEO 1450 VP scanning electron microscope (Carl Zeiss) at 10 kV acceleration voltage and a working distance of 7 mm. In this study, the cells were scored as positive or negative for filopodial spikes. Filopodia-positive cells were defined as having more than 10 thin processes beyond the cellular edge of the plasma membrane [90]. Quantitative analyses were performed in a blinded manner from 100 individual cells per each group and from three experimental preparations.

### Preparation of cell lysates

The cells were collected by centrifugation at 1000 × *g* for 5 min (4 °C), washed with PBS containing bacitracin (100 μg/ml), PMSF (0.1 mM), pepstatin A (1.0 μg/ml) and leupeptin (2.0 μg/ml), pH 7.4, and centrifuged again. The pellet was treated with lysis buffer (PBS, containing 1.0 % (v/v) Triton X-100, 0.5 % (w/v) deoxycholate and 0.1 % (w/v) SDS for 30 min at 4 °C, resuspended and centrifuged at 30000 × *g* for 15 min (4 °C).

### Protein assay

Protein was determined using the DC Protein Assay System from BioRad Laboratories according to the manufacturer instructions.

### Analysis of Src, Met, PDGFR, p42/p44 MAPK and Smad2/3 phosphorylation

#### Immunoprecipitation (IP) and Western blotting

For IP, cleared cell lysates normalized for protein content were treated with 2.0 μg anti-PDGFR antibody for 2–3 h, followed by 20 μl protein A-Sepharose beads overnight at 4 °C. Beads were collected by centrifugation, washed three times with lysis buffer containing 2.0 μg/ml leupeptin, 1.0 μg/ml pepstatin A, 100 μg/ml PMSF, 100 μg/ml aprotinin, 1.0 mM Na_3_VO_4_ and 1.0 mM NaF. Ten μl Roti®-Load were added to the beads and the samples were heated for 5 min at 95 °C.

Proteins of cell lysates (Src, Met, p42/p44 MAPK and Smad 2/3 detection) or immunoprecipitates (PDGFR detection) were subjected to 10 % SDS-PAGE and transferred to nitrocellulose membranes (BioRad Laboratories). After blocking in 1.0 % BSA/1.0 % skimmed milk for 1 h, the nitrocellulose strips were incubated overnight with the respective first antibody. Strips were washed two times with 0.05 % Tween 20 washing buffer, incubated for 45 min with the respective secondary antibody conjugated to horseradish peroxidase and washed again two times as described above. In all experiments, the immunoblots were stripped and reprobed with antibodies to total protein to confirm equal protein loading. Secondary antibodies were detected by using the chemiluminescence (ECL) Western blotting detection system (Amersham) and exposure to Kodak X-Omat films.

Immunoreactive bands for phosphorylated forms of Src (p-Src), Met (p-Met), PDGFR (p-PDGFR),p42/p44 MAPK (p-p42/p44 MAPK), Smad2 and Smad3 (p-Smad2/3) as well as Src (t-Src), total Met (t-Met), total PDGFR (t-PDGFR), total p42/p44 MAPK (t-p42/p44 MAPK), and β-actin were quantified using the image processing program Image J 1.43 (National Institutes of Health, Bethesda, Maryland, USA).

### Proteome profiler arrays

LX-2 cells, serum-starved for 17 h, were treated with PAR_2_-AP, 2-furoyl-LIGRLO-NH_2_, trypsin or vehicle for 24 h. For determination of the relative levels of different proteins in the supernatant, proteome profiler arrays [Human Angiogenesis Array Kit, Catalog Number ARY007 (allows to simultaneously detect simultaneously the relative changes of 55 angiogenesis-associated cytokines: Activin A, ADAMTS, Angiogenin, Angiopoietin-1, Angiopoietin-2, Angiostatin/Plasminogen, Amphiregulin, Artemin, Tissue Factor/Factor III, CXCL16, DPPIV/CD26, EGF, EG-VEGF Endoglin/CD105, Endostatin/Collagen XVIII, Endothelin, FGF acidic, FGF basic, FGF-4, FGF-7/KGF, GDNF, GM-CSF, HB-EGF, HGF, IGFBP-1, IGFBP-2, IGFBP-3, IL-1 beta, CXCL8/IL-8, LAP (TGF-beta 1), Leptin, CCL2/MCP-1, CCL3/MIP-1 alpha, MMP-8, MMP-9, NRG1-beta 1, Pentraxin 3, PD-ECGF, PDGF-AA, PDGF-AB/PDGF-BB, Persephin, CXCL4/PF4, P*l*GF, Prolactin, Serpin B5/Maspin, Serpin E1/PAI-1, Serpin F1/PEDF, TIMP-1, TIMP-4, Thrombospondin-1, Thrombospondin-2, uPA, Vasohibin, VEGF, VEGF-C) and Human Protease Array Kit, ARY021 (allows to simultaneously detect the relative changes of 35 proteinases: ADAM8, Cathepsin X/Z/P, MMP-7, ADAM9, DPPIV/CD26, MMP-8, ADAMTS, Kallikrein 3/PSA, MMP-9, ADAMTS13, Kallikrein 5, MMP-10, Cathepsin A, Kallikrein 6, MMP-12, Cathepsin B, Kallikrein 7, MMP-13, Cathepsin C/DPPI, Kallikrein 10, Neprilysin/CD10, Cathepsin D, Kallikrein 11, Presenilin-1, Cathepsin E, Kallikrein 13,Proprotein, Convertase 9, Cathepsin L, MMP-1, Proteinase 3, Cathepsin S, MMP-2, uPA/Urokinase, Cathepsin V, MMP-3) R&D Systems, Minneapolis, MN 55413, USA] were used according to the manufacturer’s instructions. Briefly, nitrocellulose membranes were blocked for 1 h and then incubated with 500 μl of supernatant (24 h after treatment with PAR_2_-AP or vehicle) overnight at 4 °C. After washing, the membranes were incubated with the detection antibody cocktail for 2 h and streptavidin–HRP for 30 min. For data evaluation, the membranes were exposed to chemiluminescent reagents (ECL Plus Western Blotting Detection System; Amersham) and finally to X-ray films for 30 s. The developed films were scanned and the average pixel density of the positive signals was analysed using the analysis software Image J 1.43 (National Institutes of Health, Bethesda, Maryland, USA). All images from the arrays were normalized by subtracting the background and inverted to eliminate the background differences. To measure the pixel density, a fixed size rectangular box was generated around each dot blot/band and the pixel density was measured. The same sized rectangular box was used for all bands in all arrays performed. For analysis, the pixel densities of the negative control on each array were subtracted from the pixel densities obtained from each band on the array. The data was further converted and normalized into fold change in expression by dividing the pixel densities of each band by the average pixel densities of the Streptavidin-HRP reference spots (R) located at the three corners of each array.

### Stimulation of Hep3B cells with LX-2 cell conditioned media and cell counting

Hep3B cells were seeded in 48-well plates (2 × 10^4^/well). On the next day, the growth medium was removed and replaced by 500 μl of cleared culture supernatant conditioned for 48 h by LX-2 cells transfected with either control siRNA, PAR_1_ siRNA or PAR_2_ siRNA (see above: “Transient transfection of LX-2 cells with PAR_2_ siRNA and TGF-β stimulation”). Following incubation with the LX-2 conditioned media for 72 h, the medium was removed, centrifuged to collect non-adherent cells and the resuspended pellets combined with the detached adherent cells from the respective well. Then, viable and dead cells were counted by trypan blue dye exclusion test.

### Cell migration assay

LX-2 cell migration was measured using a 48-well boyden-chamber (NeuroProbe, Inc., Gaithersburg MD, USA). 51 μl of the cell suspension (4 × 10^4^ cells in RPMI-1640) with or without inhibitor were placed in each upper chamber well and 27 μl of cell culture medium containing the chemoattractant or vehicle in each lower well. Then, incubation for 6 h at 37 °C in a humidified incubator with 5.0 % CO_2_ was performed to allow cell migration through a porous polycarbonate filter (6.5 mm in diameter, 8.0 μm pore size) precoated with collagen (0.35 %). After the incubation period, the filter was removed, and its upper side was wiped gently with a cotton tip swab to remove non-migrated cells. The migrated cells were fixed on the lower surface of the membrane with 96 % ethanol, stained with Giemsa solution, and counted under a Zeiss Axiolab microscope. Data were acquired from three independent experiments, involving octuplicate measurements for each condition.

### Estimation of cell viability

For testing the effect of inhibitors on LX-2 cell viability, cells were seeded into 24-well plates. Following treatment with the respective inhibitor for 24 h, viable and non-viable cells were distinguished by trypan blue dye exclusion method and counted manually. In addition, this test was used to monitor the viability of LX-2 cells from cultures used for animal experiments and that of Hep3B cells incubated with LX-2 culture supernatants.

### Animal model

Five to six week-old, male *SCID* mice (BALB/cJHAN®-Prkdc^*scid*^) were purchased from Harlan Laboratories Ltd. (Venray, Germany) and were handled under governmental and institutional rules and regulations after approval by the respective authorities. Animals were housed under specific pathogen-free conditions with a 14 h light/12 h dark cycle and received food and tap water ad libitum.

Mice were randomised into 5 groups, each group was consisted of 8 animals. Different cell preparations (cell viability > 95 %, estimated with trypan blue exclusion test) were suspended in 200 μl DMEM and subcutaneously injected at the right flank of the mice under general anesthesia using diethylether [group 1: 5 × 10^5^ LX-2 (wild type) cells, group 2: 10^5^ Hep3B cells; group 3: 10^5^ Hep3B cells plus 5 × 10^5^ LX-2 (wild type) cells, group 4: 10^5^ Hep3B cells plus 5 × 10^5^ LX-2 (non-target control gene knockdown) cells, group 5: 10^5^ Hep3B cells plus 5 × 10^5^ LX-2 (PAR_2_ gene knockdown) cells]. Sixteen days after xenotransplantation, micro-computed tomography (micro CT) analyses were performed under isofluorane anesthesia (CT Imaging, Erlangen, Germany). For 3D reconstruction of the tumours the software “Imalytics” (Philips GmbH, Aachen, Germany) was used. Subsequently, tumours were prepared and macroscopically evaluated. The apparent tumour volume was calculated using the formula, tumour volume (mm^3^) = (length × width^2^)/2. All tissues were fixed with paraformaldehyde and embedded in paraffin according a standard protocol and routinely stained with hematoxylin and eosin for histological examination. For immunohistochemical staining, sections (5.0 μm thickness) cut from paraffin blocks were mounted to glass slides and stained with the selected markers (monoclonal mouse anti human cytokeratin antibody, clone MNF 116; monoclonal mouse anti human Ki67 antigen, clone MIB-1, DAKO Deutschland GmbH, Hamburg, Deutschland; monoclonal rat anti Mouse CD31 (PECAM-1) antibody (DIA310, clone SZ31, Dianova GmbH, Hamburg, Deutschland).

The stained slides were scanned and digitally converted into virtual slides using the Hamamatsu NDP slide scanner (Hamamatsu Nanozoomer 2.HT) and its viewing platform (NDP.Viewer).

### Statistical analysis

The data were analysed using SPSS 13 for Windows computer program (SPSS Inc, Chicago, Illinois, USA). Statistical analyses were performed using non-parametric Mann Whitney *U* test. A *p* value <0.05 was considered to be significant.

## Abbreviations

GPCR, G protein-coupled receptor; G protein, heterotrimeric guanyl nucleotide-binding protein; HCC, hepatocellular carcinoma; HSC, hepatic stellate cell; MAPK, mitogen-activated protein kinase; Met, hepatocyte growth factor (HGF) receptor; MMP, matrix metalloproteinase; NO, nitric oxide; PAR-AP, PAR-activating peptide; PDGFR, platelet derived growth factor receptor; RTK, receptor tyrosine kinase; Src, non receptor tyrosine kinase; uPA, urokinase-type plasminogen activator

## Additional files

Additional file 1: Figure S1. PAR_2_ expression localizes to the plasma membrane. Immunolocalization of PAR_2_ on LX-2 cell plasma membrane by high-resolution field emission scanning electron microscopy. PAR_2_ was labeled in LX-2-wt cells with the monoclonal anti-PAR_2_ antibody SAM-11 in combination with a secondary antibody conjugated to immunogold. Backscatter electron image of the cellular surface of LX-2 cells with immunogold-labeled PAR_2_ complexes (20 nm gold particles, arrowheads). (TIF 210 kb) Additional file 2: Figure S2. The effect of PAR_2_-AP on [Ca^2+^]_i_ mobilisation in LX-2 cells is dose-dependent and can be blocked by the PAR_2_ antagonist GB 88. A and B) LX-2-wt cells grown on Lab Tek chambered borosilicate cover glass were loaded with fluo-4-AM as outlined in the in Method section. For calcium measurements, an inverted confocal laser scanning microscope LSM 510 was used and fluorescence was monitored at 488 nm. A) Dose-response relationship of PAR_2_-AP-induced calcium mobilisation. LX-2 cells were stimulated with PAR_2_-AP for 15 s at indicated concentrations. Data represent the mean ± SD from calcium measurements in 20 individual cells and are representative for 5 independent experiments. B) GB 88 blocks the calcium signal induced by PAR_2_-AP, but is unable to inhibit the effect of PAR_1_-AP on [Ca^2+^]_i_ mobilisation in LX-2 cells. LX-2 cells were preincubated with PAR_2_ antagonist GB 88 (10 μM) for 1 h and subsequent stimulated with PAR_2_-AP (10 μM) and PAR_1_-AP TFLLRN-NH_2_ (100 μM). The arrows indicate the time of addition of PAR_2_-AP and PAR_1_-AP (data are representative for 8 independent experiments). (TIF 19 kb)
