# Supplementary figures and images for: Proteinase-activated receptor 2 (PAR2) in hepatic stellate cells – evidence for a role in hepatocellular carcinoma growth in vivo
Source: Mol Cancer. 2016 Jul 29;15:54. doi: 10.1186/s12943-016-0538-y (PMC4966804; doi:10.1186/s12943-016-0538-y)

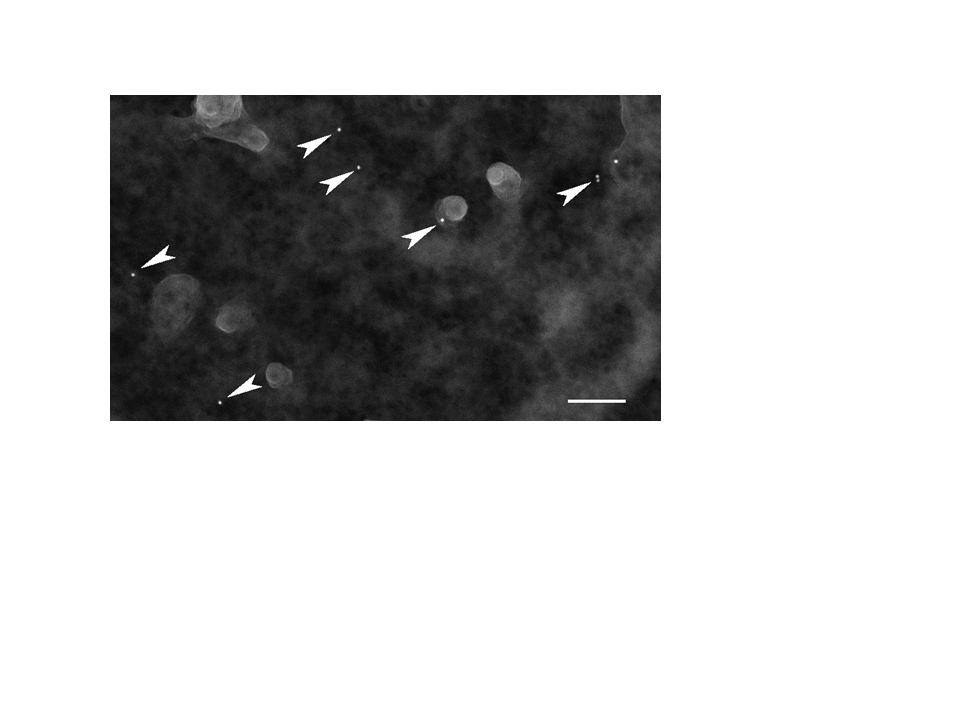

Supplement: Additional file 1: Figure S1. — PAR2 expression localizes to the plasma membrane. Immunolocalization of PAR2 on LX-2 cell plasma membrane by high-resolution field emission scanning electron microscopy. PAR2 was labeled in LX-2-wt cells with the monoclonal anti-PAR2 antibody SAM-11 in combination with a secondary antibody conjugated to immunogold. Backscatter electron image of the cellular surface of LX-2 cells with immunogold-labeled PAR2 complexes (20 nm gold particles, arrowheads). (TIF 210 kb) [file 12943_2016_538_MOESM1_ESM.tif]

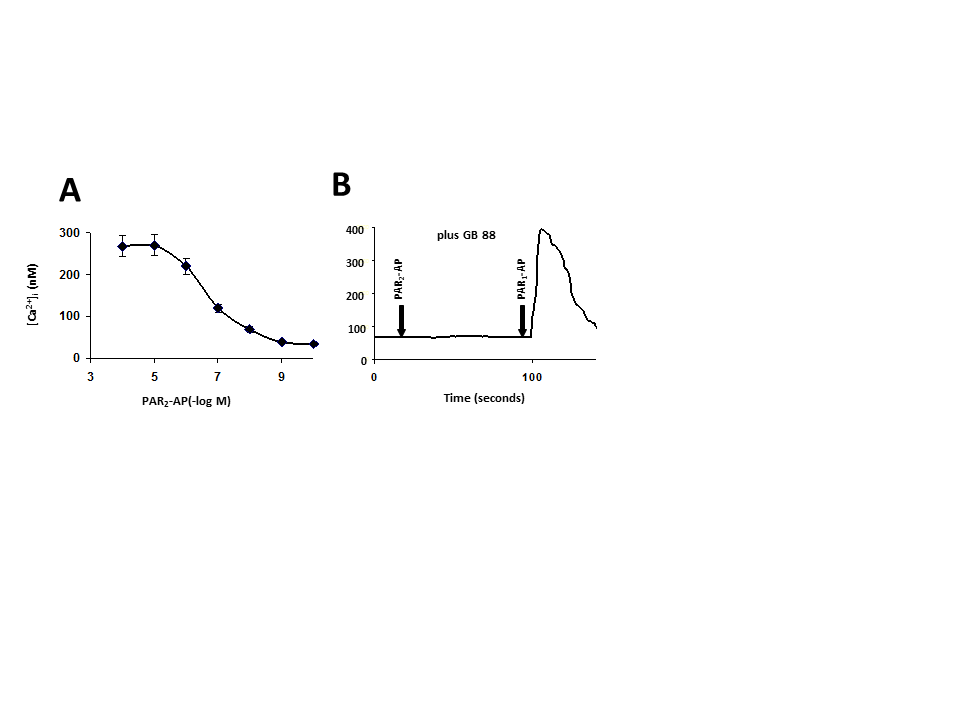

Supplement: Additional file 2: Figure S2. — The effect of PAR2-AP on [Ca2+]i mobilisation in LX-2 cells is dose-dependent and can be blocked by the PAR2 antagonist GB 88. A and B) LX-2-wt cells grown on Lab Tek chambered borosilicate cover glass were loaded with fluo-4-AM as outlined in the in Method section. For calcium measurements, an inverted confocal laser scanning microscope LSM 510 was used and fluorescence was monitored at 488 nm. A) Dose-response relationship of PAR2-AP-induced calcium mobilisation. LX-2 cells were stimulated with PAR2-AP for 15 s at indicated concentrations. Data represent the mean ± SD from calcium measurements in 20 individual cells and are representative for 5 independent experiments. B) GB 88 blocks the calcium signal induced by PAR2-AP, but is unable to inhibit the effect of PAR1-AP on [Ca2+]i mobilisation in LX-2 cells. LX-2 cells were preincubated with PAR2 antagonist GB 88 (10 μM) for 1 h and subsequent stimulated with PAR2-AP (10 μM) and PAR1-AP TFLLRN-NH2 (100 μM). The arrows indicate the time of addition of PAR2-AP and PAR1-AP (data are representative for 8 independent experiments). (TIF 19 kb) [file 12943_2016_538_MOESM2_ESM.tif]
